# A Danger-Theory-Based Immune Network Optimization Algorithm

**DOI:** 10.1155/2013/810320

**Published:** 2013-02-13

**Authors:** Ruirui Zhang, Tao Li, Xin Xiao, Yuanquan Shi

**Affiliations:** ^1^College of Computer Science, Sichuan University, Chengdu 610065, China; ^2^College of Computer Science, Huaihua University, Huaihua 418000, China

## Abstract

Existing artificial immune optimization algorithms reflect a number of shortcomings, such as premature convergence and poor local search ability. This paper proposes a danger-theory-based immune network optimization algorithm, named dt-aiNet. The danger theory emphasizes that danger signals generated from changes of environments will guide different levels of immune responses, and the areas around danger signals are called danger zones. By defining the danger zone to calculate danger signals for each antibody, the algorithm adjusts antibodies' concentrations through its own danger signals and then triggers immune responses of self-regulation. So the population diversity can be maintained. Experimental results show that the algorithm has more advantages in the solution quality and diversity of the population. Compared with influential optimization algorithms, CLONALG, opt-aiNet, and dopt-aiNet, the algorithm has smaller error values and higher success rates and can find solutions to meet the accuracies within the specified function evaluation times.

## 1. Introduction

In the practice of engineering, there are a wide variety of complex optimization problems to be solved, such as multimodal optimization, high-dimensional optimization, and dynamic optimization of time-varying parameters. These problems are manifested in the form of minimization of energy consumption, time, or risk, or maximization of the quality or efficiency, and usually can be expressed by getting the maximum or minimum of multivariable functions with a series of equations and (or) inequality constraints. In order to solve such problems, optimization theories and technologies have been rapidly developed, and its impact on society is also increasing.

Current research focus of optimization algorithms is evolutionary computation methods represented by genetic algorithms (GAs) [1–3]. The genetic algorithm simulates the biological evolution process, is a random search optimization method, and shows excellent performance in solving typical problems. Although GA has characteristics of global search and probabilistic choice, the performance of GA is sensitive to some key parameters which are crossover rate and mutation rate. Moreover, it is difficult for GA to solve multimodal function optimization due to its random crossover pairing mechanism. So, on one hand, researchers hope to make continuous improvements on existing genetic algorithms, and on the other hand, they try to build new algorithm models based on new biological theories.

Artificial immune system (AIS) is one of bionic intelligent systems inspired by biological immune system (BIS), and is new frontier research in artificial intelligence areas. The study of AIS has four major aspects, including negative selection algorithms (NSAs), artificial immune networks (AINEs), clonal selection algorithms (CLONALGs), the danger theory (DT), and dendritic cell algorithms (DCAs) [4]. It cannot only detect and eliminate nonself-antigens regarded as illegal intrusions, but also has the evolutionary learning mechanism [5–7]. There have been a great progress by applying the artificial immune to optimization problems, and many research papers have been sprung up. In artificial immune optimization algorithms, solutions to optimization problems which are to be solved and are usually expressed as high-dimensional functions are viewed as antigens, candidate solutions are viewed as antibodies, and qualities of candidate solutions correspond with affinities between antibodies and antigens [8, 9]. The process of seeking feasible solutions is the process of immune cells recognizing antigens and making immune responses in the immune system. The following works are typical. de Castro and Fernando proposed the basic structure named CLONALG [10] of function optimization and pattern recognition based on the clonal selection mechanism. Halavati et al. [11] added the idea of symbiosis to CLONALG. This algorithm is initialized with a set of partially specified antibodies, each with one specified property, and then the algorithm randomly picks antibodies to add to an assembly. This work showed better performance than CLONALG. de Castro and Von Zuben proposed an optimized version of aiNet [12], named opt-aiNet [9]. This algorithm introduces the idea of network suppression to CLONALG and can dynamically adjust the population size, having strong multivalued search capabilities. The work in [13] presented an algorithm called dopt-aiNet to suit the dynamic optimization. This algorithm introduces a line search procedure and two mutation operators, enhances the diversity of the population, and refines individuals of solutions.

Existing artificial immune optimization algorithms have maintained many merits of BIS, such as fine diversity, strong robustness, and implicit parallelism, but also reflect a number of shortcomings, such as premature convergence and poor local search ability [14, 15]. By introducing the danger theory into the optimization algorithm and integrating the clonal selection theory and the immune network theory, this paper proposes a danger-theory-based immune network optimization algorithm, named dt-aiNet. The main contributions of this paper are (1) introducing the danger theory into the optimization algorithms by simulating the danger zone and danger signals; (2) giving a new antibody concentration mechanism.

The remainder of this paper is organized as follows. The principles of artificial immune theories and influential artificial immune based optimization algorithms are described in Section 2. The flow description and optimization strategies of dt-aiNet are described in Section 3. The computational complexity, convergence, and robustness analyses of dt-aiNet are presented in Section 4. The effectiveness of dt-aiNet is verified using typical problems in Section 5. Finally, the conclusion is given in the last section.

## 2. Related Works

In this section, three artificial immune theories being adopted in this paper are introduced, including the clonal selection, the immune network, and the danger theory. And three influential artificial immune based optimization algorithms, including CLONALG, opt-aiNet, and dopt-aiNet which are to be compared with the proposed algorithm in the experiments are described.

### 2.1. Artificial Immune Theories

From the humoral immune response in the biological immune mechanism, the main idea of the clonal selection [5–7, 16] is that, when immune cells are stimulated by antigens, clonal proliferation occurs, which result in a large number of clones, and then these clones differentiate into effect cells and memory cells through the high-frequency variation. In the process of proliferation, effect cells generate a large number of antibodies, and then the antibodies duplicate and mutate to make affinities gradually increase and eventually reach affinity maturation. The clonal selection theory simulates the process of evolution of immune cells, which can learn and memorize the modes of antigens. In optimization algorithms, we simplify the concept of immune cells and use antibodies to represent a variety of immune cells. Antibodies evolve through the clonal selection theory, which means search in the solution space.

The main idea of the immune network [5–7, 17] is that, when antibodies recognize invasive antigens, a variety of antibodies constitute a dynamic network through interactions between themselves. The immune system is viewed as a mutual influential and mutual restricted network. The network can maintain a balance according to the immune regulation mechanism. When similarity between antibodies is higher, the network will produce inhibition. When similarity is low, the network will produce stimulus. So, the network can maintain population diversity and equilibrium, and ultimately becomes stable, composed of a variety of memory cells. The theory is an important complement and development to the clonal selection theory. In the optimization algorithms, the concept that the immune network can delete redundant solutions and maintain the balance of global and local search is used.

The danger theory [18] proposed by Matzinger indicated that the key why the immune system is able to distinguish the nonself-antigens and self-antigens is that these nonself-antigens make the body produce biochemical reactions different from natural rules and the reactions will make the body produce danger signals of different levels. So, the immune system produces danger signals based on the environmental changes and then leads to the immune responses. In essence, the danger signal creates a danger zone around itself and immune cells within this danger zone will be activated to participate in the immune response. Compared with the clonal selection theory and the immune network theory, the danger theory introduces the environmental factors of the body, describes some important characteristics of the biological immune system, and explains some immune phenomena which the traditional immune theory cannot explain, such as autoimmune diseases. Therefore, through combination of the clonal selection theory and the immune network theory, the danger theory can simulate the biological immune mechanism more completely and accurately. The theory is a new addition to artificial immune algorithms. The danger theory is introduced in this paper to express the ambient environmental state of antibodies, which can better simulate the biological immune system, maintain the population diversity, and accelerate the convergence of the algorithm. In danger theory, there are not any specific definitions of danger signals. So, using the danger theory is crucial to defining the suitable danger signals and danger zones according to the actual situation.

### 2.2. Influential Optimization Algorithms

CLONALG [10] proposed by de Castro simulates the activation process of immune cells. Only those immune cells that can recognize antigens split and amplify. Clones of immune cells with high affinity are more, and the variation rate is small; clones of cells with low affinity are less, and the variation rate is large. This algorithm searches for the global optimal solutions through the cloning and high-frequency variation of immune cells, which makes full use of the diversity mechanism in the immune system. This algorithm is simple, and the disadvantage is the premature convergence [8, 14].

opt-aiNet [9] introduces the concept of immune network based on the clonal selection theory into optimization problems. This algorithm is described in Algorithm 1.

opt-aiNet includes two loops. At first, the algorithm enters into the first loop. Implant a specific number of antibodies (real-valued vectors) in the definition domain of the objective function, constituting the artificial immune network. Then, the algorithm enters into the second loop. In order to obtain the local optimal solution, perform the clonal selection to every antibody in the network. The process continues until the average fitness of the population is close to that of the previous generation, which means that the network is stabilized. Then, the algorithm jumps out of the second loop. Antibodies in the network interact with each other, and the network suppression occurs. At last, randomly introduce new antibodies. Repeat the process until the termination conditions are met. Due to the nested loops, the algorithm increases unnecessary function evaluation times. The algorithm maintains the diversity of the population, but has disadvantages of slow convergence and low search accuracy [8, 14].

dopt-aiNet extends opt-aiNet to deal with time-varying fitness functions [13]. This algorithm introduces a line search procedure called golden section and two mutation operators, which are one-dimensional mutation and gene duplication. The golden section is to choose the best step size of mutation. The one-dimensional mutation performs similarly to the traditional Gaussian mutation but only for one direction at a time. In the operation of gene duplication, a randomly chosen element (coordinate) is copied to another element, simulating the chromosome behavior in the evolution of species. This algorithm increases the search accuracy, but the two mutation operations waste too much function evaluation times, which makes the algorithm converge slowly.

## 3. Description of the Proposed Algorithm

This section describes the basic idea of dt-aiNet. The flow of the algorithm is described in Section 3.1. The simulation of optimization algorithm for the immune system is introduced in Section 3.2. And the optimization strategies of dt-aiNet are introduced in Section 3.3. These strategies are complementary to each other and are applied in the process of the algorithm.

### 3.1. Flow Description

In this paper, the danger theory is introduced into the optimization algorithm and the clonal selection theory and the immune network theory are integrated. All the antibodies which interact with each other form the immune network. First, the algorithm defines the danger zone to calculate danger signals for each antibody and then adjusts antibodies' concentrations through its own danger signals. Second, the algorithm performs the clonal proliferation operation, generating clone groups by duplicating a certain number of random antibodies, and then mutates each clone, but keeps the parent antibody. Third, the algorithm selects the antibody with highest fitness which is in the parent antibody's danger zone and selects antibodies with higher fitness than the parent antibody which are not in the parent antibody's danger zone. Fourth, the algorithm adds randomly generated antibodies to adjust the population size, recalculates danger signals for all antibodies, and then removes antibodies whose concentration equals to zero. All the individuals in the population constitute the immune network which improves the affinities of the population in constant evolution. The network makes antibodies with low concentration and low affinity dead, and survival antibodies are viewed as memory individuals. When the number of memory individuals does not change, these individuals are the optimization solutions of the multimodal function. Therefore, the algorithm composes of seven elements, danger signals and concentrations calculation, clonal selection (*T*_*s*_), clonal proliferation (*T*_*c*_), hypermutation (*T*_*m*_), clonal suppression (*T*_*cs*_), network suppression (*T*_*ns*_), and population updating (*T*_*u*_). The termination conditions are that the function evaluation times (FEs) reach the maximum or the function error of the found optimal solution reaches or is less than a specified value (Algorithm 2).

### 3.2. Representations of Antibodies, Antigens, and Affinities

The optimization function is expressed as *P* = min⁡*f*(*x*). The variable *x* = (*x*_1_, *x*_2_,…, *x*_*n*_) ∈ *R* ^*n*^ is the decision variable, and the variable *n* is the variable dimension. The function min represents obtaining minimum of function *f*(*x*), and we can also obtain maximum of function *f*(*x*). The algorithm uses real coding. Assumed that *Ab* represents the antibodies set which is also the population of the network, *Ag* represents the antigens set. For the rest of the paper, population always means the collection of antibodies. So, antibody *Ab*_*i*_ and antigen *Ag*_*j*_ are  *n*-dimensional real vectors, and *i*,  *j* are natural integers. The antibody population is of *R* ^*n*^ scale. So the optimization problem can be transformed to min⁡{*f*(*Ab*_*i*_), *Ab*_*i*_ ∈ *R* ^*n*^}  or  max⁡{*f*(*Ab*_*i*_), *Ab*_*i*_ ∈ *R* ^*n*^}.

The affinity between antibody and antigen is the binding strength between antibody and antigen, which is the solution fitness to the problem. It is expressed by affinity(*Ab*_*i*_), and is the normalized representation of function value *f*(*Ab*_*i*_). The affinity of *Ab*_*i*_ is calculated according to (1)

(1) $$\begin{array}{c} \text{affinity}\left(Ab_{i}\right)=\{\begin{array}{cc} \frac{f\left(Ab_{i}\right)-f_{\min}}{f_{\max}-f_{\min}}, & P=\max f\left(x\right),\\ 1-\frac{f\left(Ab_{i}\right)-f_{\min}}{f_{\max}-f_{\min}}, & P=\min f\left(x\right), \end{array} \end{array}$$

where *f*(*Ab*_*i*_) is the function value of antibody *Ab*_*i*_, *f*_min⁡_ is the minimum of the current population, and *f*_max⁡_ is the maximum of the current population.

The affinity between antibody and antibody represents the similarity degree between the two antibodies and is expressed by affinity(*Ab*_*i*_, *Ab*_*j*_). For real coding, it is usually related to the distance between the two antibodies and is calculated as follows:

(2) $$\begin{array}{c} \text{affinity}\left(Ab_{i},Ab_{j}\right)=\frac{1}{\text{dis}\left(Ab_{i},Ab_{j}\right)}, \end{array}$$

where dis is the Euclidean distance between antibody *Ab*_*i*_ and antibody *Ab*_*j*_ and is expressed as follows:

(3) $$\begin{array}{c} \text{dis}\left(Ab_{i},Ab_{j}\right)=\sqrt{\sum_{k=1}^{n}{\left(Ab_{ik}-Ab_{jk}\right)}^{2}}. \end{array}$$

### 3.3. Optimization Strategies

This section describes some of the steps in the process of the algorithm, which are different from the influential artificial immune based optimization algorithms.

#### 3.3.1. Danger Zone and Danger Signals

Because danger signals are associated with the environment, we use the proximity measurement to simulate the danger zone. The concentrations of antibody populations in the danger zone reflect the environment condition for the optimization problem. According to the danger theory [18], if an antigen *Ag*_*i*_ necrotizes, the nearby area *Ag*_*i*_-centered will become a danger zone *D*(*Ag*_*i*_). Because antigens are invisible for the optimization problem, we assume that each antibody is the peak point, and the vicinity around the peak is the danger zone. The danger zone is defined as follows:

(4) $$\begin{array}{c} D\left(Ab_{i}\right)=\left\{Ab_{j}\;|\;\text{dis}\left(Ab_{i},Ab_{j}\right)<r_{\text{danger}}\right\}, \end{array}$$

where *r*_danger_ is the danger zone radius, and the value is related to the intensity of the peak points.

Interactions between antibodies within *Ab*_*i*_'s danger zone are *Ab*_*i*_'s environmental state. Then, the danger signal function  *ds*  is defined by (5). This function takes the concentration con(*Ab*_*j*_) of antibody *Ab*_*j*_ whose affinity is greater than affinity(*Ab*_*i*_) in *Ab*_*i*_'s danger zone and the distance dis(*Ab*_*i*_, *Ab*_*j*_) between *Ab*_*i*_ and *Ab*_*j*_ as inputs and then produces the danger signal of antibody *Ab*_*i*_:

(5) ds(Abi)  =∑Abj∈D(Abi)∩affinity(Abj)>affinity(Abi)con(Abj)            ·(rdanger−dis(Abi,Abj)),

where con is the antibody concentration. In the population, only if antibody *Ab*_*j*_ is within the danger zone of antibody *Ab*_*i*_, and the affinity between *Ab*_*j*_ and antigens is greater than the affinity between *Ab*_*i*_ and antigens, antibody *Ab*_*j*_ will exert an influence on antibody *Ab*_*i*_. The higher the concentration of *Ab*_*j*_ is, the greater the impact on the environment of antibody *Ab*_*i*_ is. The closer *Ab*_*i*_ and *Ab*_*j*_ are, the greater the impact on the environment of antibody *Ab*_*i*_ is.

#### 3.3.2. Concentration Calculation

The antibody concentration is dynamic and is related to the danger signal of the antibody and the affinity between the antibody and antigens. These two factors are the main reasons for the dynamically changing of antibody concentration.

When the surroundings change, the antibody concentration will change. If the danger signal of an antibody is not zero; that is to say, there are better solutions around the antibody and the danger signal will inhibit the antibody, the concentration of the antibody will decay with the evolution. The greater the danger signal is, the greater the impact on the environment of the antibody is. When the surroundings do not change, the antibody is in a relatively stable environment; that is to say, there are not better solutions around the antibody. So, the antibody is regarded as a candidate peak point, and the concentration of the antibody will increase with the evolution. 

The affinity between the antibody and antigens will affect the antibody's concentration as well. The greater the affinity is, the better the fitness of the antibody as a solution is. When the antibody is regarded as a candidate peak point, the increment of the antibody's concentration will be proportional to the affinity. When the danger signal of the antibody exists, the attenuation of the antibody's concentration will be inversely proportional to the affinity.

The concentration con(*Ab*_*i*_) of antibody *Ab*_*i*_ is calculated according to (6). In the equation, con(*Ab*_*i*_) depends on the iteration. The variable *t* represents evolution generation, and *t* + 1 means the next generation after *t*. So, con(*Ab*_*i*_)_*t*_ means the concentration of antibody *Ab*_*i*_ at generation  *t*:

(6) con(Abi)t+1={con(Abi)t(1+exp⁡(affinity(Abi)0.25))               ds(Abi)=0,con(Abi)t(1−ln⁡(1+affinity(Abi))affinity(Abi))−ds(Abi)                   ds(Abi)>0.

For the initial population, each antibody is set an initial concentration con_0_. When the danger signal of the antibody exists, the antibody's concentration will gradually decrease and ultimately to zero. When it does not exist, the antibody's concentration will gradually increase and up to 1. Therefore, con(*Ab*_*i*_)∈[0, 1]. Danger signals provide the changes of concentrations of antibodies a baseline and maintain the diversity of the population.

#### 3.3.3. Mutation Operation

The mutation operation simulates high-frequency variation mechanism in the immune response. And this operator generates antibodies with higher affinities and enhances the diversity of antibody population. The algorithm of opt-aiNet [9] adopts Gaussian variation, and the related formulas are as follows:

(7) $$\begin{array}{c} c^{\prime }=c+\alpha N\left(0,1\right),\\ \alpha =\left(\frac{1}{\beta }\right)\exp\left(-f^{*}\right), \end{array}$$

where  *c*′  is a mutated cell  *c*,  *N*(0,1)  is the Gaussian random variable with mean 0 and deviation of 1, and f* is the fitness of an individual normalized in the interval [0,1].  *β*  is the control parameter to adjust the mutation range and is an user-specified value in the algorithm of opt-aiNet.

**Figure 1:**
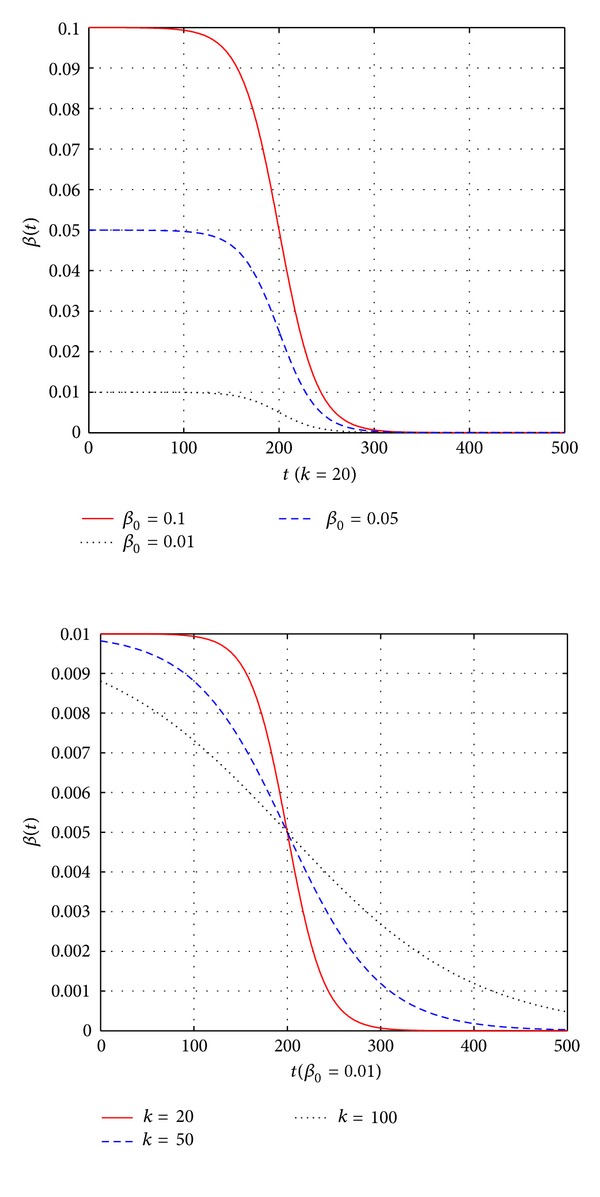
Changing curves of  *β*.

There are certain shortcomings in this method. For different functions,  *β*  is difficult to determine. In the search process, if  *β*  is too large, individuals will search with higher probability, which is more conducive to global search and leads to a slow rate of convergence. If  *β*  is too small, individuals will search with smaller probability, which is more conducive to local search and makes the algorithm searching around the local minimums, impossible to escape from the local minima and result in precociousness. Therefore, this paper adopts dynamic self-adaptive  *β*, and the mutation mechanism is expressed as follows:

(8) $$\begin{array}{c} Ab_{i}\left(t+1\right)=Ab_{i}\left(t\right)+\alpha N\left(0,1\right),\\ \alpha =\beta \left(t\right)\exp\left(-\text{affinity}\left(Ab_{i}\right)\right),\\ \beta \left(t\right)=\frac{\beta_{0}}{1+\exp\left(\left(t-t_{0}\right)/k\right)}, \end{array}$$

where  *t*  is the number of iteration times. *Ab*_*i*_(*t*) means the antibody *Ab*_*i*_ at generation  *t*, and *Ab*_*i*_(*t* + 1) means the antibody *Ab*_*i*_ at generation *t* + 1. In the initial stage of the algorithm,  *β*  is large, and the algorithm approaches toward the peak points with higher probability, which speeds up the convergence rate. When the algorithm iterates a certain number of times,  *β*  becomes small, and the algorithm searches in the neighborhood of the peak points, which improves the accuracy of solutions. Because affinity(*Ab*_*i*_)∈[0,1], exp⁡(−affinity(*Ab*_*i*_))∈[0.3679,1]. *β*_0_ is the control parameter and determines the range of *β*, and *β* ∈ [0, *β*_0_].  *k*  is the regulation parameter and adjusts the rate of change of exp⁡(*t* − *t*_0_). *t*_0_ is the demarcation point of *β* changes, that is, global search with large probability and local search with small probability. When *t* < *t*_0_, *β* ∈ [*β*_0_/2, *β*_0_], and the algorithm should search into the neighborhood of peak points. When *t* > *t*_0_, *β* ∈ [0, *β*_0_/2], and the algorithm starts to do a small-scale search near the peaks.  Figure 1 shows the changing curves of  *β*  under different initial values of *k* and *β*_0_. In the first chart,  *k*  is stable, and *β*_0_ varies between 0.1, 0.01, and 0.05. As can be seen, the range of  *β*  is  [0, *β*_0_]. So, we need to select the appropriate *β*_0_. In the second chart, *β*_0_ is 0.01, and *k* varies between 20, 50, and 100. The larger the value of *k* is, the more evidently  *β*  changes. The smaller *k* is, the smaller the change rate of  *β*  is.

#### 3.3.4. Suppression Operation

In artificial immune optimization algorithms, the suppression operations are divided into two kinds, which are clonal suppression and network suppression.

Performing the clone operation to every antibody in the population will produce clone groups. Then, variations of clone groups will create antibodies with higher affinity. The clone suppression means retaining antibodies with higher affinity from clone groups, and giving up the rest of the clone individuals. In opt-aiNet, clonal suppression means selecting the antibody with highest affinity from the temporary set which is composed of the parent antibody and its clonal group to join the network. dt-aiNet still chooses this way to add antibodies into the network, and meanwhile selects antibodies into the network which have higher affinity than the parent antibody and are not in the parent antibody's danger zone. So, clonal suppression operation *T*_*cs*_ can be expressed as follows:

(9) $$\begin{array}{c} T_{cs}\left({Ab}_{\left\{i\right\}}\right)={{Ab}_{\left\{i\right\}}}^{\prime }+{{Ab}_{\left\{i\right\}}}^{\prime \prime }, \end{array}$$

where *Ab*_{*i*}_ is the collection of antibody *Ab*_*i*_ and its clonal group. *Ab*_{*i*}_′ and *Ab*_{*i*}_′′ are expressed by (10) and (11). In (10), it selects antibodies with highest affinity in the parent antibody's danger zone. In (11), it selects antibodies with higher affinity than that of the parent antibody and not in the parent antibody's danger zone. After the two selection operations, clonal suppression operation retains better antibodies and discards the other ones:

(10) Ab{i}′={Ab{i}k ∣ affinity(Ab{i}k)=max⁡(affinity(Abj))  ∩Abj∈Ab{i}∩Ab{i}k∈D(Abi)},

(11) Ab{i}′′={Ab{i}k ∣ affinity(Ab{i}k)>affinity(Abi)  ∩Ab{i}k∉D(Abi)}.

Network suppression operation simulates the immune network regulation principle, which reduces the redundant antibodies and eliminates similar solutions. In dt-aiNet, this operation deletes antibodies with concentrations equaling to zero. An antibody's concentration is zero indicates that the danger signals of this antibody always exist, and there are better individuals around this antibody. This antibody is redundant. Network suppression operation *T*_*ns*_ can be expressed as follows:

(12) $$\begin{array}{c} T_{ns}\left(Ab\right)=Ab-\left\{Ab_{i}\;|\;\text{con}\left(Ab_{i}\right)=0\right\}. \end{array}$$

## 4. Algorithm Analyses

This section analyzes the algorithm from three aspects, including the computational complexity, the convergences and the robustness.

### 4.1. Computational Complexity Analysis

**Theorem 1**

The computational complexity of dt-aiNet is *O*(*t*′ · *N* ^2^ · *n*) or *O*(*t*′ · *N* · *Nc* · *n*), where *t*′ is the total number of iterations, *N* is the population size, *n* is the dimension of the problem to be solved, and *Nc* is the max number of clones which an antibody generates.

**Proof**

As shown in the algorithm flow, dt-aiNet consists of six major components: the clonal selection operation, the cloning operation, the mutation operation, the suppression operations, the population updating operation, and the danger signals and concentrations adjusting operations. In iteration  *t*, the number of calculation times of the clonal selection operation is *N*. The number of the calculation times of the cloning operation does not exceed *N* · *Nc*. The number of calculation times of the mutation operation does not exceed *N* · *Nc* · *n* because each dimension of a vector needs to mutate. The number of calculation times of the suppression operations does not exceed *N* · *Nc*.

Supposing the population size is *N*_1_, *N*_1_ ≥ *N*, and *N*_1_ is related to *N*, after the suppression operations. The calculation number of the population updating operation is  *d*% · *N*_1_ · *N*_1_, where *d* is the percentage of population updating and is a user-specified value. The calculation number of the danger signals and concentrations adjusting operations is *N*_1_ · (*N*_1_ − 1) · *n*, where we first calculate the danger zone of each antibody, then compute the danger signal of each antibody, and at last adjust the concentration of each antibody. In iteration *t*, the total number *g*(*t*) of calculation times meets

(13) g(t)≤N+N·Nc+N·Nc·n+N·Nc +d%·N1·N1+N1·(N1−1)·n.

Therefore, if the total number of iterations is *t'*, the computational complexity of algorithm is *O*(*t*′ · *N* ^2^ · *n*) or *O*(*t*′ · *N* · *Nc* · *n*). This expression shows that the time complexity of the algorithm is related to the population size  *N*.

Similarly, the calculation complexities of CLONALG and opt-aiNet can be analyzed.  Table 1 shows the contrasts of the calculation complexities of the three algorithms. In the case of a certain dimension, reducing the population size can greatly reduce the complexity of the algorithm.

### 4.2. Convergence Analysis

From the running mechanism of dt-aiNet, each generation of the population consists of two parts. One is the memory antibodies from the previous generation, and another is the new antibodies randomly added. Antibodies with higher affinities from the mutation operation are mainly in the neighborhood of the parent antibody. After the clonal suppression operation, population affinities will be higher than those of the previous generation. The antibodies with higher affinities will change the surrounding environments and then make danger signals of antibodies with lower affinities in the danger zone stronger and their concentrations lower. As the generation increases, if antibodies with lower affinities cannot escape from the danger zone under strengthened danger signals, their concentrations will decay to zero and then they will die. Antibodies with high affinities will retain in the memory population due to the unchanged environments. In this mechanism, antibodies in the memory population basically have high affinities and are peak points. It will be ensured that new antibodies randomly added to the population in each generation are not in the danger zone of memory antibodies. So, they will develop a new search space, and then the algorithm will eventually find all the peaks with the evolution.

Same as before, we assume that *t* is the number of generation. So, *Ab*(*t*) represents the population  *Ab*  at generation *t*. Due to the state of population, *Ab*(*t* + 1) is only related to that of the previous generation *Ab*(*t*), and has nothing to do with those of the past generations, the entire population sequences {*Ab*(*t*)} constitute a random process of the Markov chain [19].

**Theorem 2**

For any distribution of the initial population, dt-aiNet is the weak convergence of probability, that is to say,

(14) $$\begin{array}{c} \underset{t\to \infty }{\lim}P\left(Ab\left(t\right)\cap Ab^{*}\neq \emptyset \right)=1, \end{array}$$

where Ab* is a set which contains the optimal solution.

**Proof**

Known from the total probability formula,

(15) P(Ab(t+1)∩Ab∗=∅) =P(Ab(t)∩Ab∗=∅)  ·(1−P(Ab(t+1)∩Ab∗≠∅ ∣ Ab(t)∩Ab∗=∅))  +P(Ab(t)∩Ab∗≠∅)  ·P(Ab(t+1)∩Ab∗=∅ ∣ Ab(t)∩Ab∗≠∅).

After operations of selection, clone, mutation, and suppressions, affinities of population  *Ab*(*t*)  will arise, That is to say,

(16) $$\begin{array}{c} \text{affinity}\left(Ab\left(t+1\right)\right)\geq \text{affinity}\left(Ab\left(t\right)\right). \end{array}$$

So,

(17) $$\begin{array}{c} P\left(Ab\left(t+1\right)\cap Ab^{*}=\emptyset \;|\;Ab\left(t\right)\cap Ab^{*}\neq \emptyset \right)=0. \end{array}$$

From the above equation, we have

(18) P(Ab(t+1)∩Ab∗=∅) =P(Ab(t)∩Ab∗=∅)  ·(1−P(Ab(t+1)∩Ab∗≠∅ ∣ Ab(t)∩Ab∗=∅)).

Suppose *Ab*_*i*_ ∈ Ab*, *Ab*_*i*_ ∈ *Ab*(*t* + 1), and *Ab*_*i*_ ∉ *Ab*(*t*), then,

(19) P(Ab(t+1)∩Ab∗≠∅ ∣ Ab(t)∩Ab∗=∅)  =P(Tc,m,s,cs,ns,u(Ab(t))=Ab(t+1))  ≥P(Tm(Abj)=Abi)=ε,

where *T*_*c*,*m*,*s*,*cs*,*ns*,*u*_ means these operations including clone, mutation, selection, suppression, and updating.

Known from the induction,

(20) $$\begin{array}{c} P\left(Ab\left(t\right)\cap Ab^{*}=\emptyset \right)\leq {\left(1-\varepsilon \right)}^{t}. \end{array}$$

So,

(21) $$\begin{array}{c} \underset{t\to \infty }{\lim}P\left(Ab\left(t\right)\cap Ab^{*}=\emptyset \right)=0; \end{array}$$

that is,

(22) $$\begin{array}{c} \underset{t\to \infty }{\lim}P\left(Ab\left(t\right)\cap Ab^{*}\neq \emptyset \right)=1-\underset{t\to \infty }{\lim}P\left(Ab\left(t\right)\cap Ab^{*}=\emptyset \right)=1. \end{array}$$

### 4.3. Robustness Analysis

The algorithm contains a number of parameters. Most of them have little effect on the search performance and can be set conventionally. But the two parameters *k* and *t*_0_ are more critical and will affect the algorithm performance.  *k*  is the adjustable parameter of mutation rate  *β*  and decides the change rate of  *β*. *t*_0_ is demarcation point of changes of  *β*, that is, cutoff point of the global search with a high probability and the local search with a small probability. There are two evaluation indicators of robustness measurement, which are the relationship between the convergence probability and the parameter set (*k*, *t*_0_) and the relationship between the average evaluation number of the function and the parameter set (*k*, *t*_0_). 

Here are three definitions to more clearly explain the evaluation indicators [20].

**Definition 3 (successful test)**

Given the parameters and the max iterative times to be allowed, if the function error between the optimal solution and the best solution gained from running the algorithm is not greater than  *ε*, the test is successful, and then the algorithm stops.

**Definition 4 (convergence probability)**

It means the success ratio in tests of *m* times.

**Definition 5 (the average number of evaluation times)**

Given the parameters and the max iterative times to be allowed, the average number of evaluation times is the average times of computing the objective function in tests of *m* times.

We choose the ninth function  *F*_9_(*x*)  defined in the work [21] as the testing function. And the work in [21] provides the optimization accuracy  1*e* − 2  for this function. Here is the definition of this function:

(23) $$\begin{array}{c} F_{9}\left(x\right)=\sum_{i=1}^{D}\left({z_{i}}^{2}-10\cos\left(2\pi z_{i}\right)+10\right)+f_{\text{bia}{\text{s}}_{9}}, \end{array}$$

where *z* = *x* − *o*, *x* = [*x*_1_, *x*_2_,…, *x*_*D*_], *D* is the dimension, and *x* ∈ [−5,5]^*D*^. *o* is the extreme point of the function, and *o* = [*o*_1_, *o*_2_,…, *o*_*D*_]. *F*_9_(*o*) = *f*_bias_9__ = −330.

**Figure 2:**
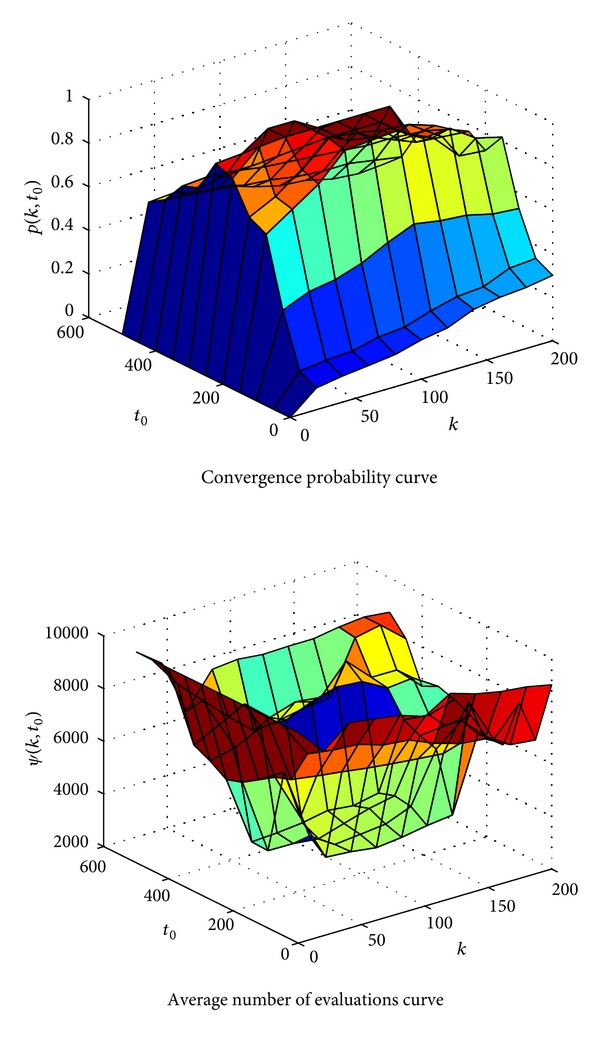
Charts of changes of parameter robustness.

Given *ε* = 0.01 and *m* = 25, this function includes a large number of local optimal solutions and a global optimal solution. These solutions are relatively evenly distributed, and there are many local optimal solutions near the global optimal solution. The minimum of the function is −330. We select this function for the robustness test, mainly because this function is relatively more complex, and its features are poor, and general intelligent algorithms are difficult to get satisfactory results.  Figure 2 shows the relationships with the convergence probability *p*(*k*, *t*_0_) and the relationships with the average evaluation number *ψ*(*k*, *t*_0_).

As can be seen from Figure 2, when *k* → 0 and *t*_0_ → 0, the convergence probability is basically zero, and the average evaluation number is close to the maximum evaluation number of 10000. This range is the nonconvergence zone. Because the variation is very small and almost negligible in this range, only immune selection operation and population updating operation contribute to the search process, and the search process is completely random. Thus, the algorithm is basically impossible to guarantee the convergence. When *k* → 200 and *t*_0_ → 500, the convergence probability is greater than zero but small, and the average evaluation number is close to the maximum evaluation number of 10000 as well. This range is the danger zone. In this range, the mutation rate is large, and the algorithm is easy to jump out of the neighborhood of peak points. So, it will search for a long time to get the optimal solution. When *k* and *t*_0_ is in the middle range, the convergence probability approaches 1, and the average evaluation number is close to 3400, which is the minimum evaluation number to find the optimal solution. So, for these two parameters, we should choose values of the middle range.

## 5. Experiments

This section applies the algorithm to the benchmark functions, which run in 2-dimensional spaces and 10-dimensional spaces. The selection of functions and the evaluation criteria of algorithms are described in Section 5.1. The experimental results are shown in Section 5.2 as well as comparisons with the other three artificial immune based optimization algorithms.

### 5.1. Function Selection and Evaluation Criteria

For that the performance evaluation criteria of optimizing algorithms are not uniform, Suganthan et al. [21] jointly published the report about problem definitions and evaluation criteria on real-parameter optimization in the 2005 IEEE Congress on Evolutionary Computation. In this report, 25 benchmark functions are given, and a common termination criterion, size of problems, initialization scheme, and so forth are specified. We choose *F*_2_, *F*_4_, *F*_9_, and *F*_12_ and related evaluation criteria, including function error values of the optimal solution, the gained peak numbers, success rates, and convergence graphs to assess the quality and the efficiency of the algorithms. 

The termination conditions are that FEs reach *n*∗10^4^  (*n*  is the dimension), or the function error value of the found optimal solution reaches or is less than the required function errors [21].

We select the influential optimization algorithms based on artificial immune to do the experiments, including CLONALG, opt-aiNet, and dopt-aiNet. The accuracies of the optimization functions are shown in Table 2. The parameters of the four algorithms are as follows:

(24) $$\begin{array}{c} F_{2}\left(x\right)=\sum_{i=1}^{D}{\left(\sum_{j=1}^{i}z_{j}\right)}^{2}+f_{\text{bia}{\text{s}}_{2}}, \end{array}$$

where *z* = *x* − *o*, *x* = [*x*_1_, *x*_2_,…, *x*_*D*_], *x* ∈ [−100,100]^*D*^, *F*_2_(*o*) = *f*_bias_2__ = −450

(25) $$\begin{array}{c} F_{4}\left(x\right)=\left(\sum_{i=1}^{D}{\left(\sum_{j=1}^{i}z_{j}\right)}^{2}\right)*\left(1+0.4\left|N\left(0,1\right)\right|\right)+f_{\text{bia}{\text{s}}_{4}}, \end{array}$$

where *z* = *x* − *o*, *x* = [*x*_1_, *x*_2_,…, *x*_*D*_], *x* ∈ [−100,100]^*D*^, *F*_4_(*o*) = *f*_bias_4__ = −450

(26) $$\begin{array}{c} F_{9}\left(x\right)=\sum_{i=1}^{D}\left({z_{i}}^{2}-10\cos\left(2\pi z_{i}\right)+10\right)+f_{\text{bia}{\text{s}}_{9}}, \end{array}$$

where *z* = *x* − *o*, *x* = [*x*_1_, *x*_2_,…, *x*_*D*_], *x* ∈ [−5,5]^*D*^, *F*_9_(*o*) = *f*_bias_9__ = −330

(27) $$\begin{array}{c} F_{12}\left(x\right)=\sum_{i=1}^{D}{\left(A_{i}-B_{i}\left(x\right)\right)}^{2}+f_{\text{bia}{\text{s}}_{12}},\\ A_{i}=\sum_{j=1}^{D}\left(a_{ij}\sin\alpha_{j}+b_{ij}\cos\alpha_{j}\right),\\ B_{i}\left(x\right)=\sum_{j=1}^{D}\left(a_{ij}\sin x_{j}+b_{ij}\cos x_{j}\right),\;\text{for}\;\;i=1,\ldots ,D, \end{array}$$

where *A* and *B* are two  *D*∗*D*  matrices, *a*_*ij*_  and  *b*_*ij*_ are integer random numbers in the range [−100,100], *α* = [*α*_1_, *α*_2_,…, *α*_*D*_], *α*_*j*_ are random numbers in the range [−*π*, *π*], *x* = [*x*_1_, *x*_2_,…, *x*_*D*_], *x* ∈ [−*π*,*π*]^*D*^, *F*_12_(*α*) = *f*_bias_12__ = −460.

The parameters of dt-aiNet are *N* (initial population size) = 50, *k* (regulation of mutation rate) = 20, *t*_0_ (demarcation point of mutation rate) = 200, *β*_0_ (range of mutation rate) = 0.01, con_0_ (initial concentration) = 0.5, *Nc* (number of clones) = 10, *r*_danger_ (radius of danger zone) = 0.1, and *d*% (percentage of updating population) = 0.3. 

The parameters of CLONALG are  *N*  (initial population size) = 50,  *β*  (mutation rate) = 0.01, and  *Nc*  (number of clones) = 10.

The parameters of opt-aiNet are  *N*  (initial population size) = 50,  *Nc*  (number of clones) = 10,  *β*  (mutation rate) = 100, *σ*_*s*_ (network suppression threshold) = 0.2 or 0.05, and  *d*%  (percentage of updating population) = 0.4.

The parameters of dopt-aiNet are  *N*  (initial population size) = 50,  *Nc*  (number of clones) = 10,  *β*  (mutation rate) = 100, *σ*_*s*_  (network suppression threshold) = 0.5, and  *d*%  (percentage of updating population) = 0.4.

### 5.2. Results of Performance Tests

The algorithms run in 2-dimensional space and 10-dimensional space for the above functions in order to accurately assess the performances.

Table 3 shows the results of performing 25 times for the four algorithms in 2-dimensional space, including function error values (*f* − f*) of the optimal solution and peak numbers, where values in brackets are variances. From Table 3, we can see that errors of opt-aiNet are lower than those of CLONALG and dopt-aiNet, and errors of dt-aiNet are lower than those of opt-aiNet. Although dopt-aiNet has local search operation, the two new mutation operations, one-dimensional mutation and gene duplication, take up too much evaluation times; so the algorithm usually cannot find the optimal solution yet when reaches the maximum number of evaluation times. In addition, for the two unimodal functions, *F*_2_ and *F*_4_, dt-aiNet can only find the optimal solution, while CLONALG, opt-aiNet, and dopt-aiNet not only find the optimal solution, but also some redundancy solutions.

Table 4 shows the results of performing 25 times for the four algorithms in 2-dimensional space, including success rates and success performances. Known from the work [21], the optimization success rate is defined by *Success Rate = successful runs/total runs*, and the optimization success performance is defined by *Success Performance = mean (FEs for successful runs)∗(total runs)/(successful runs).* It can be seen from Table 4 that only dt-aiNet can find the solution which meets the accuracies when limiting the maximum number of function evaluation times.

**Figure 3:**
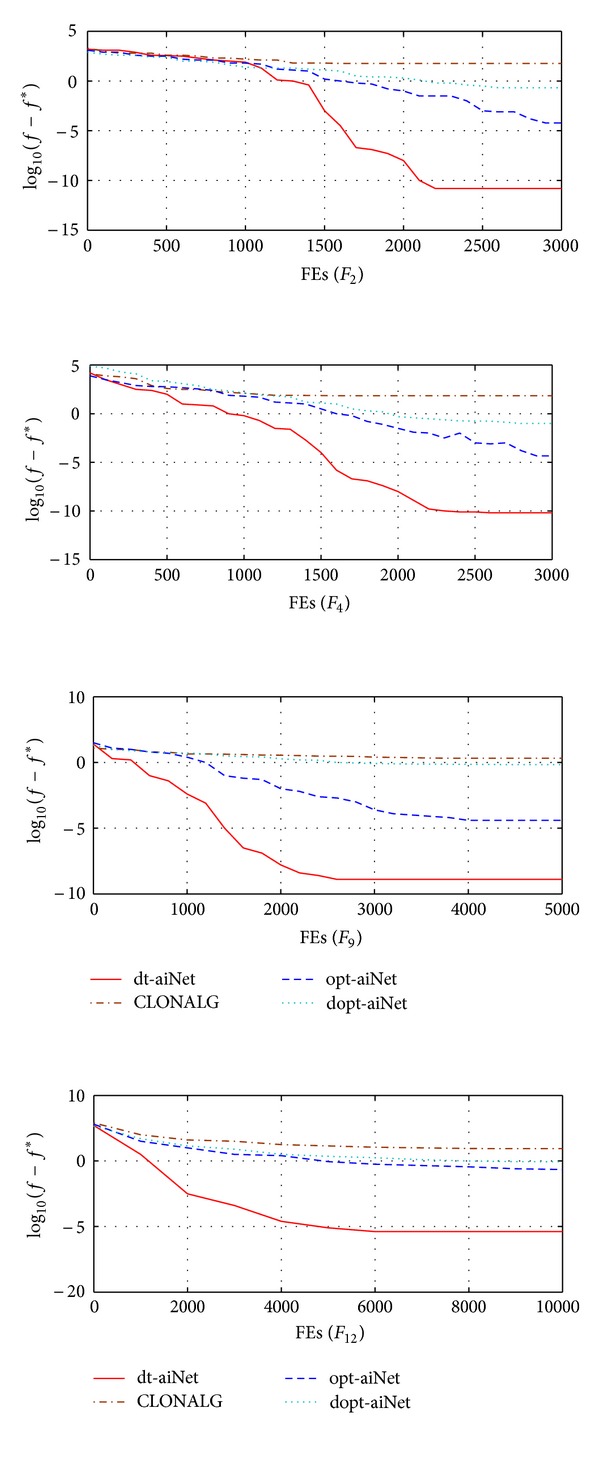
Convergence graphs in 2-dimensional spaces.

Figure 3 shows the convergence graphs in 2-dimensional space of the four algorithms. As can be seen, after initial populations are randomly generated, the convergence curve of each algorithm continues to lower with the evolution. CLONALG is easily trapped in local minima. Opt-aiNet maintains a good diversity of the population, but converges slowly due to the nested loops and increasing unnecessary function evaluation times. dopt-aiNet can find solutions with greater accuracies because of the local search operation, but it wastes a large number of function evaluation times for performing the two mutation operations to the memory population and the nonmemory population. So dopt-aiNet converges more slowly. dt-aiNet maintains a better diversity of the population by extracting the environmental information and mutating in a dynamic rate and makes the population quickly converge to the optimal solution.

Tables 5 and 6 show the results of performing 25 times for the four algorithms in 10-dimensional space. Seen from the tables, dt-aiNet still possesses preferable optimization performances in high-dimensional space and is better than CLONALG, opt-aiNet, and dopt-aiNet. In addition, the average function error values and variances are relatively stable and are able to maintain a high level in the 25 times of independently running.

**Figure 4:**
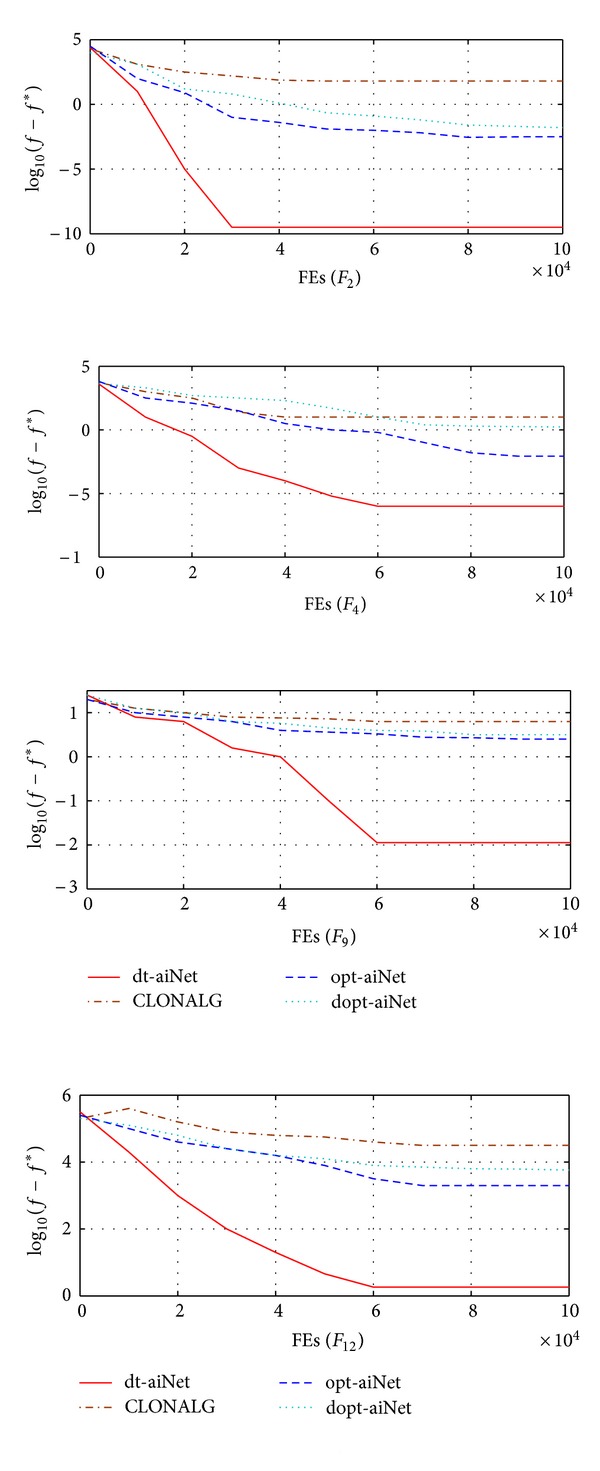
Convergence graphs in 10-dimensional spaces.

Figure 4 shows the convergence graphs in 10-dimensional space of the four algorithms. As seen from the graphs, dt-aiNet still possesses preferable optimization performances with the increase of dimensions and is better than CLONALG, opt-aiNet, and dopt-aiNet.

## 6. Conclusions

This paper proposes a danger-theory-based immune network optimization algorithm, named dt-aiNet, for solving multimodal optimization problems. In order to increase the solution quality and the population diversity, the proposed algorithm introduces the danger theory into the optimization algorithms and integrates the clone selection theory and the immune network theory. It simulates the danger zones and the danger signals and adopts concentrations to comprehensively evaluate antibodies. Experimental results show that compared with influential optimization algorithms based on artificial immune, including CLONALG, opt-aiNet, and dopt-aiNet, the proposed algorithm has smaller error values and higher success rates and can find solutions to meet the accuracies within the specified FEs. However, the algorithm cannot apply to any kind of optimization problems, and with the increase of dimension, the success rates of the algorithm are not always 100%. The next steps will be improving the efficiency of the algorithm in the high-dimensional space and extending the application scopes, such as dynamic optimization, combinatorial optimization, and constrained optimization.

## Figures and Tables

**Algorithm 1:**
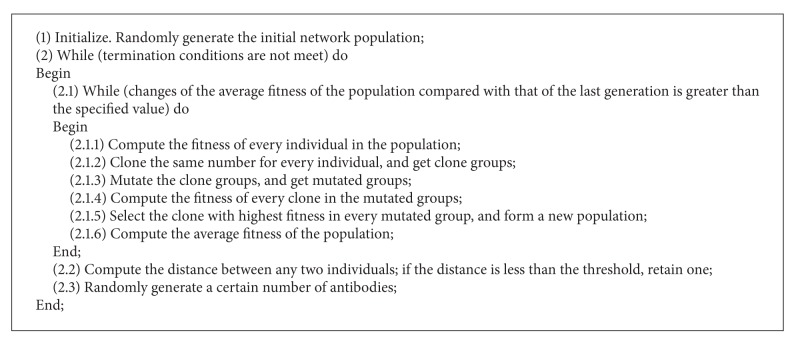
The description of opt-aiNet.

**Algorithm 2:**
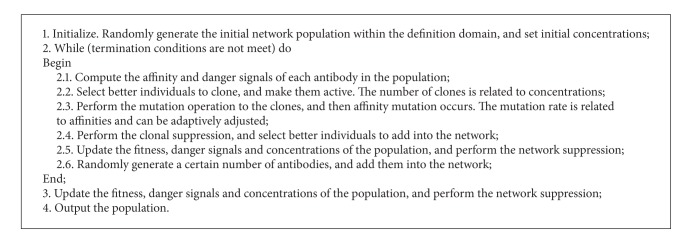
The description of dt-aiNet.

**Table 1:** Calculation complexities of the algorithms.

Algorithms	Complexities
CLONALG	*O(t′* · *N* · *Nc* · *n)* [10]
opt-aiNet	*O(t′* · *N* ^2^ · *n)* or *O(t′* · *N* · *Nc* · *n)* [9]
dt-aiNet	*O(t′* · *N* ^2^ · *n)* or *O(t′* · *N* · *Nc* · *n) *

**Table 2:** Accuracies of functions.

Functions	Accuracies
*F*_2_	−450 + 1*e* − 6
*F*_4_	−450 + 1*e* − 6
*F*_9_	−330 + 1*e* − 2
*F*_12_	−460 + 1*e* − 2

**Table 3:** Results (errors) in 2-dimensional spaces.

		Function errors of the optimal solution	Number of peaks
	dt-aiNet	1.62∗10^−11^ (2.1∗10^−11^)	1 (0)
*F*_2_	CLONALG	5.78∗10^1^ (3.34∗10^1^)	1 (1.46)
	opt-aiNet	6.01∗10^−5^ (4.61∗10^−5^)	5 (1.42)
	Dopt-aiNet	2.13∗10^−1^ (4.5∗10^−1^)	2.41 (1.2)

	dt-aiNet	5.86∗10^−11^ (1.25∗10^−11^)	1 (0.2)
*F*_4_	CLONALG	6.93∗10^1^ (3.38∗10^1^)	3.6 (2.21)
	opt-aiNet	4.57∗10^−5^ (4.32∗10^−5^)	5.8 (2.2)
	dopt-aiNet	1.03∗10^−1^ (5.81∗10^−1^)	3.69 (1.3)

	dt-aiNet	1.2∗10^−9^ (1.03∗10^−9^)	82.54 (8.22)
*F*_9_	CLONALG	2.12∗10^0^ (4.58∗10^0^)	45.6 (20.28)
	opt-aiNet	3.99∗10^−5^ (2.47∗10^−5^)	60.11 (23.87)
	dopt-aiNet	6.87∗10^−1^ (3.9∗10^−1^)	32.09 (12.7)

	dt-aiNet	1.68∗10^−11^ (1.06∗10^−11^)	7.22 (0.43)
*F*_12_	CLONALG	7.56∗10^1^ (4.35∗10^1^)	4.6 (3.10)
	opt-aiNet	5.01∗10^−2^ (2.23∗10^−2^)	5 (1.43)
	dopt-aiNet	7.61∗10^−1^ (5.84∗10^−1^)	8.67 (1.33)

**Table 4:** Results (success rates) in 2-dimensional spaces.

	dt-aiNet		CLONALG		opt-aiNet		dopt-aiNet	
	Success rates	Success performance	Success rates	Success performance	Success rates	Success performance	Success rates	Success performance
*F*_2_	100%	2.209∗10^3^	0%	—	0%	—	0%	—
*F*_4_	100%	2.576∗10^3^	0%	—	0%	—	0%	—
*F*_9_	100%	3.413∗10^3^	0%	—	0%	—	0%	—
*F*_12_	100%	5.278∗10^3^	0%	—	0%	—	0%	—

**Table 5:** Results (errors) in 10-dimensional spaces.

		Function errors of the optimal solution	Number of peaks
	dt-aiNet	7.52∗10^−10^ (1.84∗10^−10^)	1 (0)
*F*_2_	CLONALG	9.74∗10^1^ (2.67∗10^1^)	57.80 (7.26)
	opt-aiNet	5.32∗10^−3^ (4.61∗10^−3^)	13.76 (6.81)
	dopt-aiNet	1.56∗10^−2^ (5.77∗10^−2^)	46.49 (1.33)

	dt-aiNet	9.65∗10^−7^ (3.24∗10^−7^)	1 (0.1)
*F*_4_	CLONALG	1.32∗10^1^ (5.79∗10^1^)	123.6 (11.02)
	opt-aiNet	8.68∗10^−3^ (3.54∗10^−3^)	5 (1.17)
	dopt-aiNet	7.14∗10^−1^ (2.94∗10^−1^)	12.2 (2.32)

	dt-aiNet	1.12∗10^−2^ (2.11∗10^−2^)	188.33 (0.31)
*F*_9_	CLONALG	3.17∗10^2^ (4.58∗10^2^)	45.6 (20.28)
	opt-aiNet	5.66∗10^1^ (2.47∗10^1^)	433.55 (3.43)
	dopt-aiNet	7.43∗10^1^ (2.80∗10^1^)	52.5 (4.67)

	dt-aiNet	1.83∗10^0^ (5.66∗10^0^)	193.5 (5.65)
*F*_12_	CLONALG	3.22∗10^4^ (6.43∗10^4^)	376.67 (5.19)
	opt-aiNet	2.06∗10^3^ (1.33∗10^3^)	379.43 (0.33)
	dopt-aiNet	5.69∗10^3^ (2.14∗10^3^)	41.61 (2.26)

**Table 6:** Results (success rates) in 10-dimensional spaces.

	dt-aiNet		CLONALG		opt-aiNet		dopt-aiNet	
	Success rates	Success performance	Success rates	Success performance	Success rates	Success performance	Success rates	Success performance
*F*_2_	100%	2.677∗10^4^	0%	—	0%	—	0%	—
*F*_4_	100%	5.542∗10^4^	0%	—	0%	—	0%	—
*F*_9_	100%	4.798∗10^4^	0%	—	0%	—	0%	—
*F*_12_	93%	5.415∗10^4^	0%	—	0%	—	0%	—
